# Functional Neuroimaging as a Biomarker of Non-Invasive Brain Stimulation in Upper Limb Recovery After Stroke: A Systematic Review and Narrative Discussion

**DOI:** 10.3390/biomedicines14010117

**Published:** 2026-01-06

**Authors:** Sheharyar S. Baig, Wen Hai, Mudasar Aziz, Paul Armitage, Kevin Teh, Ali N. Ali, Arshad Majid, Li Su

**Affiliations:** 1 Sheffield Institute for Translational Neuroscience, Division of Neuroscience, University of Sheffield, Sheffield S10 2HQ, UKali.ali@sheffield.ac.uk (A.N.A.);; 2 Division of Clinical Medicine, University of Sheffield, Sheffield S10 2RX, UK; 3 Department of Psychiatry, University of Cambridge, Cambridge CB2 0SP, UK

**Keywords:** stroke, arm weakness, rehabilitation, neuroimaging, functional MRI, functional near-infrared spectroscopy

## Abstract

**Introduction**: Stroke is a leading cause of adult-onset disability. Non-invasive brain stimulation (NIBS) techniques such as repetitive transcranial magnetic stimulation (rTMS), transcranial direct current stimulation (tDCS), and transcutaneous vagus nerve stimulation (tVNS) are promising adjuncts to upper limb rehabilitation. The use of functional neuroimaging through task functional MRI (fMRI) or functional near-infrared spectroscopy (fNIRS) allows the visualisation of cortical activation patterns associated with stroke-related impairment and recovery. The present study comprehensively reviews the evidence base for the effects of NIBS on clinical and functional neuroimaging outcomes after stroke. **Methods**: Systematic searches were carried out in MEDLINE and EMBASE via Ovid. Inclusion criteria were clinical trials of adults with stroke and arm weakness undergoing NIBS, with clinical measures of arm function and neuroimaging outcome measures that included either task fMRI or task fNIRS. Two authors independently carried out study screening, risk of bias assessments, and data collection for clinical and neuroimaging outcomes pre- and post-intervention. **Results**: A total of 17 studies (12 rTMS, 5 tDCS), including 495 participants, met the inclusion criteria. Fifteen studies used task fMRI and four used task fNIRS. Improvements in arm-related motor activity were observed following both rTMS and tDCS. Most studies reported increased activation in ipsilesional sensorimotor areas alongside reductions in contralesional activation. **Discussion**: rTMS and tDCS may improve upper limb recovery in people with stroke. The increase in the laterality index towards activation of the ipsilesional hemisphere suggests that these NIBS techniques may facilitate neural reorganisation and restoration of motor networks in the affected hemisphere.

## 1. Introduction

Stroke is a leading cause of adult-onset disability [1]. While physiotherapy and occupational therapy can improve upper limb recovery, arm weakness affects up to 60% of stroke survivors who experience flaccid hemiplegia [2]. In chronic stroke (>6 months post-onset), spontaneous recovery is limited, and high doses of therapy are required to promote restoration of function [3]. As such, there is an unmet need for adjuncts to rehabilitation that potentiate neural plasticity within sensorimotor networks.

**Figure 1:**
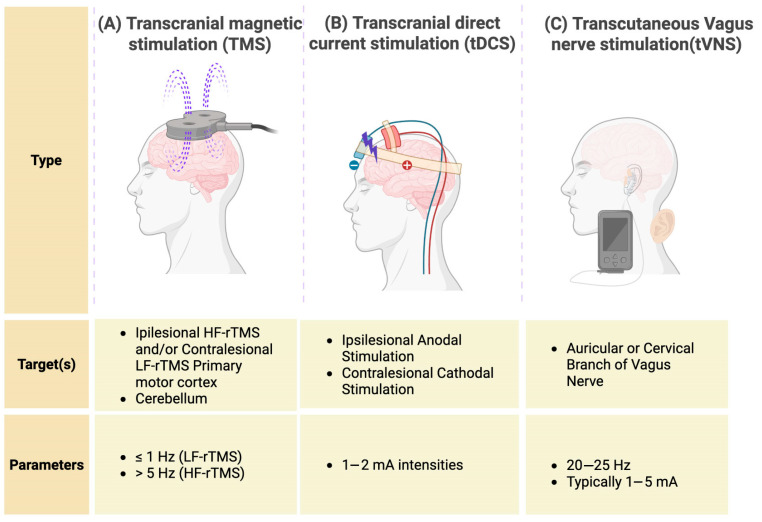
Types of non-invasive brain stimulation (NIBS) used in stroke rehabilitation. The figure illustrates the three main NIBS modalities examined in this review: (**A**) repetitive transcranial magnetic stimulation (rTMS), which uses magnetic pulses to modulate cortical excitability; (**B**) transcranial direct current stimulation (tDCS), which applies weak electrical currents via scalp electrodes; and (**C**) transcutaneous vagus nerve stimulation (tVNS), which targets the auricular or cervical branches of the vagus nerve to promote neuroplasticity. Created in BioRender. Baig, S. (2026) https://BioRender.com/9a5afy5 (acccessed on 20 December 2025).

Non-invasive brain stimulation (NIBS) techniques are emerging treatment modalities that may alter the neural response to rehabilitation. Examples of NIBS include repetitive transcranial magnetic stimulation (rTMS), transcranial direct current stimulation (tDCS), and transcutaneous vagus nerve stimulation (tVNS) (Figure 1A). rTMS has frequency-dependent effects on cortical networks [4]. High-frequency rTMS (HF-rTMS) (>1 Hz) is typically excitatory and applied to the ipsilesional motor regions, whereas low-frequency rTMS (LF-rTMS) (<1 Hz) is typically inhibitory and applied to the contralesional hemisphere to reduce its potential maladaptive inhibitory influence on ipsilesional motor activity [4]. tDCS utilises weak direct currents via scalp electrodes to influence the excitability of specific brain regions. tVNS involves activation of the cervical or auricular branches of the vagus nerve and is typically delivered prior to or during motor rehabilitation to promote task-specific plasticity [5].

Determining the efficacy of NIBS modalities on arm recovery after stroke requires large, multi-centre clinical trials in diverse stroke cohorts. Challenges for such studies include maintaining adequate recruitment and retention within rehabilitation clinical trials, the time commitment for research staff, the cost of using medical devices, and the variability in spontaneous recovery and response to rehabilitation between individuals [6,7]. Biomarkers of stroke recovery, such as functional neuroimaging, can offer insight into the mechanism of stroke recovery and provide an ancillary measurement of neural plasticity [8]. Bridging the understanding between mechanism and clinical effects in stroke recovery may provide more confidence in the potentially restorative effects of NIBS. Early observed biomarkers may also enable more economically viable clinical trials [9].

Functional MRI (fMRI) and functional near-infrared spectroscopy (fNIRS) are non-invasive tools to assess cerebral function [10,11]. They enable the assessment of cerebral haemodynamics, including blood flow and oxygenation changes at rest (resting-state connectivity) and in response to tasks (e.g., motor tasks), providing an indirect measure of neuronal activity. Task fMRI and fNIRS work on the principle of the BOLD response, whereby local neuronal activation results in initial transient oxygen consumption, a fall in oxygenated haemoglobin, and a subsequent compensatory increase in oxygenated haemoglobin in that region [11]. The changes in the magnetic and optical properties of haemoglobin at different levels of oxygen saturation allow the indirect measurement of neural activity during a task compared to rest [11]. fMRI offers greater spatial resolution and can easily be mapped onto individual patient-level structural MRI.

fNIRS offers improved comfort and movement tolerance but is limited by modest spatial resolution and a shallow penetration depth, meaning deeper structures such as the basal ganglia, thalamus, and cerebellum cannot be assessed [12].

In this study, we investigate the effects of NIBS (rTMS, tDCS, and tVNS) on clinical and functional neuroimaging outcomes in randomised controlled trials of arm recovery after stroke.

## 2. Methods

The systematic review followed PRISMA guidelines (Supplementary Table S1) [13], the protocol was registered on on the Open Science Framework (OFS) (https://osf.io/jq2mx, accessed on 28 October 2025), the code is 10.17605/OSF.IO/NH6Q3.

### 2.1. Search Strategy

We systematically searched EMBASE and MEDLINE via Ovid from the inception of the databases to 11 June 2024 (date of search) for relevant studies. The full search strategy is outlined in Supplementary Table S2. Additional studies were identified through citation searches and full-text retrieval of published conference abstracts.

### 2.2. Study Selection Criteria

To be included, studies had to fulfil the following criteria:

(1) A clinical trial of adults with stroke with arm weakness (single-arm, randomised controlled trial or crossover trial).
(2) Intervention with NIBS (rTMS, tDCS or tVNS) for greater than one session.
(3) Include a clinical assessment of arm function at baseline and post-intervention.
(4) Assess fMRI or fNIRS during upper limb motor tasks at baseline and post-intervention
(5) Be written in English.

Exclusion criteria included studies published as conference abstracts rather than full-text articles and studies that solely reported on resting state fMRI or fNIRS.

Two authors (SSB, WH) independently screened titles and abstracts, excluding studies that did not meet the inclusion criteria. Full-text articles were reviewed when necessary to determine eligibility. Differences in opinion were discussed by the reviewers, and a consensus of opinion was reached.

### 2.3. Data Collection

Two authors (SSB, WH) independently extracted data from the studies using a pre-specified data collection proforma. Extracted information included sample size, country of study, demographics, stroke subtype and timing, NIBS intervention details, concurrent therapy details, clinical outcome measures of arm function and details of task fMRI or task fNIRS results. Discrepancies were resolved by a third author (LS).

### 2.4. Risk of Bias Assessment

Risk of bias for the included RCTs was assessed using the Cochrane Risk-of-Bias Tool for Randomised Trials version 2 (RoB 2) [14] by two authors (MA, WH). Discrepancies in assessment outcome were resolved after discussion with a third author (SSB). Risk of bias for non-RCTs was assessed using the Cochrane ROBINS-I V2 tool by two authors (SSB, MA) [15]. The overall risk of bias was determined as low risk (low risk of bias in all domains), some/moderate concerns (some concerns in at least one domain but not high risk in any single domain), or high/serious risk (high risk of bias in one domain or some concerns in multiple domains).

### 2.5. Narrative Synthesis

Given heterogeneity in study designs, intervention details, outcome measures, and methods of neuroimaging analysis, a meta-analysis was not performed. A narrative synthesis was performed after grouping studies according to NIBS intervention and by functional neuroimaging modality.

## 3. Results

**Figure 2:**
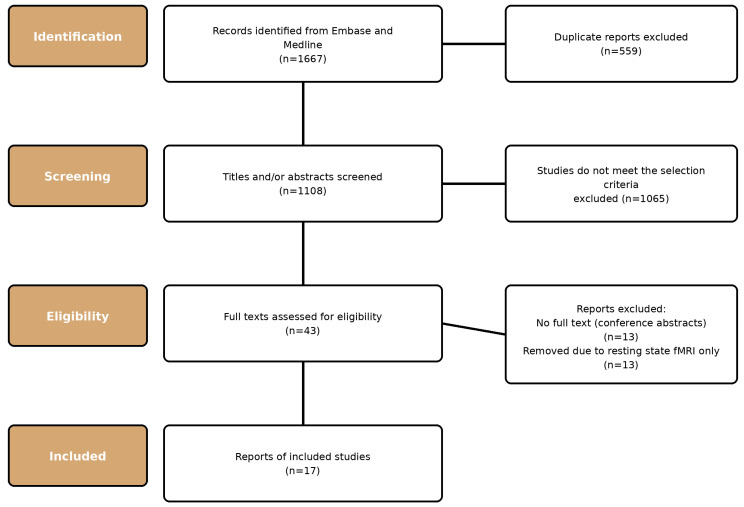
Study selection flow diagram outlining the inclusion and exclusion process.

The process of study selection is outlined in Figure 2. The search strategy yielded 1108 unique titles. A total of 17 studies, containing 495 participants, met the inclusion criteria [16,17,18,19,20,21,22,23,24,25,26,27,28,29,30,31,32].

### 3.1. Study Characteristics

**Figure 3:**
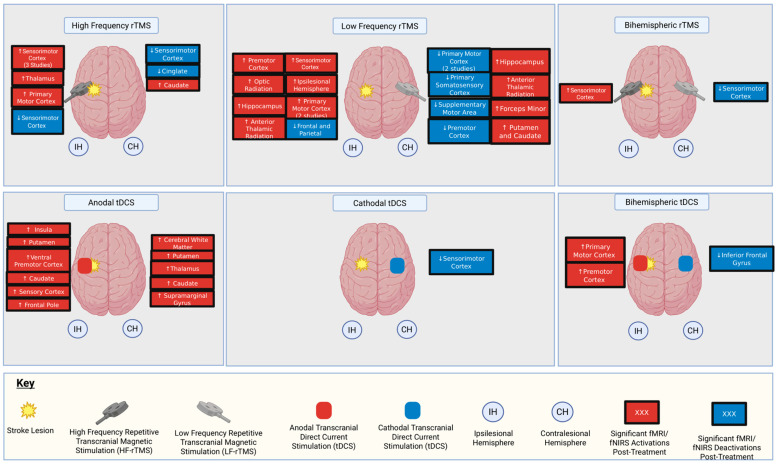
Summary of brain regions demonstrating significant post-intervention activation and deactivation across the included studies. ↑ represents statistically significant increases in task fMRI or fNIRS activation in the specified region. ↓ represents statistically significant decreases in task fMRI or fNIRS activation in the specified region. Created in BioRender. Baig, S. (2026) https://BioRender.com/qv9dlu0 (accessed on 20 December 2025).

Twelve studies involved rTMS (369 participants), and five studies used tDCS (126 participants). No studies using tVNS met the inclusion criteria. All but two studies had a majority of male participants. All but one study reported on stroke subtype [18]. Five studies included ischaemic stroke alone [18,20,21,25,32], whilst the remainder included participants with ischaemic and haemorrhagic stroke. Four studies primarily recruited participants in the subacute phase after stroke (7 days to 6 months), whereas the remainder primarily recruited people with chronic stroke (>6 months). The duration of intervention ranged from 5 days to 6 weeks. Individual study characteristics, including participant demographics, interventions, and durations, are summarised in Table 1. Brain regions demonstrating significant post-intervention activation and deactivation across the included studies are summarised in Figure 3.

### 3.2. Functional Imaging Approaches

Thirteen studies used task fMRI to assess motor-related activity (11 rTMS studies, 2 tDCS studies) while four studies used task fNIRS (2 rTMS studies, 2 tDCS studies). A variety of motor tasks were performed including sequential finger tapping, hand-grip tasks/hand flexion–extension, finger tracking tasks and bilateral motor tasks. Block lengths varied between 14 and 40 s and the number of task blocks varied between 3 and 15 repeats. Speed of movements, where specified, varied between 0.25 and 1 Hz. Neuroimaging conditions are summarised in Table 2.

A full version of this table is provided in Supplementary Table S3.

Different analytical and interpretive approaches for task fMRI were explored. These included reporting the laterality index of activation (an index of ipsilesional vs. contralesional activation), the number of active voxels pre- vs. post-intervention, the percentage increase in voxels activated in the ipsilesional hemisphere post-intervention, and reporting regions in which there was a significant change in activated voxels post-intervention. For fNIRS, the studies reported either on the laterality index or the channels that showed a significant change in activation post-intervention.

### 3.3. rTMS

Four studies reported an active treatment group targeting HF-rTMS to the ipsilesional M1 region. Additionally, one study reported intermittent theta burst stimulation (iTBS) of the ipsilesional M1 region [26]. Post-intervention, HF-rTMS or iTBS was associated with improvements in range of motion in extension, Fugl–Meyer Upper Extremity (FMA-UE) scores, Modified Ashworth Scale (MAS) scores for spasticity, and movement accuracy, but not the Jebsen–Taylor Hand Function Test (JHFT). For the RCTs with a sham control group, there were significantly greater increases in FMA-UE following HF-rTMS compared to sham stimulation [16,17,25].

Eight studies reported an active treatment group targeting LF-rTMS to the contralesional M1. Clinical improvements were seen compared to baseline in FMA-UE, Wolf Motor Function Test (WMFT) performance times, WMFT functional scores, Brunnstrom Stages of Stroke Recovery Upper Extremity (BRS-UE), and MAS at the wrist. In RCTs with a sham stimulation group, LF-rTMS was associated with improvements in Box and Block Test (BBT) time [24] and FMA-UE [24]. In a study comparing combined HF- and LF-rTMS with HF-rTMS or sham rTMS, combined rTMS resulted in greater increases in FMA-UE at 4 weeks than HF-rTMS alone or sham rTMS [16]. In a head-to-head study of HF-rTMS vs. LF-rTMS vs. sham rTMS, both active treatment groups were associated with greater increases in FMA-UE compared to sham [25].

All the studies of HF-rTMS reported changes in activation patterns compared to baseline. While some reported increases in activation in ipsilesional sensorimotor regions, including the sensorimotor cortex and thalamus [17], others reported a decrease in ipsilesional sensorimotor cortex activation [30].

For LF-rTMS, all studies showed an increase in activation in ipsilesional regions compared to baseline, or a shift in the laterality index indicating greater ipsilesional activation relative to contralesional activation. Ipsilesional regions showing increased activation compared to baseline included ipsilesional PMC, M1, SMA, optic radiation, hippocampus, and the anterior thalamic radiation. One study showed a reduction in ipsilesional activation in frontal and parietal regions [23]. Contralesional areas showing decreased activation compared to baseline included M1, S1, PMC, and SMA. For the two RCTs with a sham rTMS group, one study of LF-rTMS resulted in significantly reduced contralesional M1 activation [25] compared to sham rTMS, while another only had three participants, making it difficult to draw definitive conclusions [24].

A study using fNIRS similarly reported increased ipsilesional and reduced contralesional sensorimotor cortex activation after HF-rTMS, with a greater effect size of combined HF- and LF-rTMS vs. HF-rTMS alone. In a study comparing HF vs. LF-rTMS, HF-rTMS led to increased ipsilesional M1 and SMA activation compared to LF-rTMS and sham [25]. Similarly, LF-rTMS led to reduced contralesional M1 activity compared to HF-rTMS and sham. This is in keeping with an effect specific to the regions being stimulated or inhibited.

Correlations between clinical and neuroimaging outcomes were only reported in two studies. There was a correlation between post-intervention ipsilesional fMRI activation and motor function at longer-term follow-up (3 months) in one study of HF vs. LF-rTMS [25]. However, in this study, there was no correlation between change in motor activation pattern and clinical outcomes. Ueda et al. (2020) reported no significant relationship between change in fMRI laterality index and FMA-UE or WMFT following LF-rTMS, despite improvements in FMA-UE [23].

Two sources explicitly cited or used a specific, validated Minimal Clinically Important Difference (MCID) value to evaluate whether their reported statistical improvements represent clinically significant change [20,21]. Tosun et al. (2017) [20] used an MCID threshold of 9–10.19 points for the Upper Extremity Fugl–Meyer Assessment (UE-FMA) in subacute stroke, finding that while their control group achieved a statistically significant improvement of 4.7 points, this fell below the MCID and therefore lacked clinical relevance; conversely, their experimental groups exceeded this threshold with gains of 12.7 and 22.2 points, confirming substantial clinical benefit. Similarly, Chiu et al. (2020) [21] used a validated MCID of 4.25 points for the Fugl–Meyer motor arm score in chronic stroke, reporting that although their group median change of 2.5 points was insufficient, 43% of individual subjects achieved clinically meaningful improvement.

### 3.4. tDCS

All tDCS studies included a sham tDCS control condition. Four studies were RCTs, while one was a crossover study [31]. Two studies were of anodal (ipsilesional M1), one study was of cathodal (contralesional M1), and two studies were bihemispheric tDCS. There were no head-to-head studies comparing anodal vs. cathodal stimulation, nor bihemispheric vs. single-hemisphere stimulation. Improvements in upper limb outcome measures (FMA-UE, WMFT, BBT, and ARAT) were found in all studies. For bihemispheric tDCS, a study with a 5-day (5-session) intervention was associated with a mean 5.6-point increase in FMA-UE [32], whereas a 5-week intervention (20 sessions) was associated with a mean increase of 11.1 points [22]. The results from individual studies are summarised in Table 2.

Compared to sham tDCS, anodal tDCS led to an increase in fMRI activation in several brain regions [27]. On task-fNIRS, there was an increase in activation in a channel corresponding to the ipsilesional motor cortex, but no significant decrease in activation in contralesional motor areas following anodal tDCS [31]. For cathodal tDCS, the magnitude of contralesional motor activation decreased in 71% of participants compared to 50% in the sham intervention [18]. Bihemispheric stimulation was found to increase fMRI ipsilesional M1, PMC, and contralesional inferior frontal gyrus activation [32]. An fNIRS study of bihemispheric stimulation found increased activation in channels corresponding to the right dorsolateral prefrontal cortex, right somatosensory association cortex, and right primary motor cortex [22]. Given that lesions in this cohort were a mixture of left-sided and right-sided strokes, we cannot infer whether these changes are primarily ipsilesional or contralesional.

For cathodal stimulation, there was a significant inverse correlation between decreased contralesional activation and FMA-UE increases [18]. For bihemispheric stimulation, one study found a correlation between the laterality index in the precentral gyrus following an elbow movement task and WMFT performance improvement in the real tDCS group, but not sham [32].

### 3.5. Risk of Bias Assessment

The risk of bias assessment within and across studies is shown in Table 3 (RCTs) and Table 4 (non-RCTs). In Table 3, assessed using Cochrane’s RoB 2 tool, 7 out of 11 studies (63.6%) were rated as having a low risk of bias, while 4 studies (36.4%) showed some form of bias; notably, 3 of these (27.3% of the total) were given an overall “some” risk of bias. All studies with bias were in the rTMS group, whereas all the tDCS studies demonstrated low bias. For non-randomised trials (Table 4), the ROBINS-I v2 assessment indicated that the majority (66.7%) had an overall low risk of bias. However, all these studies exhibited some degree of bias related to outcome measurement due to the absence of detailed prespecified analyses. Additionally, most studies lacked blinded outcome assessors, leading to a moderate risk of bias in the rTMS studies. In contrast, the single tDCS study in this group showed no evidence of bias.

## 4. Discussion

This review demonstrates that non-invasive brain stimulation (rTMS and tDCS) may significantly improve upper limb function after stroke, with these clinical changes often associated with detectable shifts in motor task-related activity within sensorimotor regions of the cortex. Previous studies of rTMS and tDCS show potentially promising clinical effects in stroke survivors [33]. While many of the studies included in this review were conducted over a short time frame, changes in motor activation patterns may indicate neural organisation and plasticity that is a driver and potential biomarker of restitution of cerebral function.

The shift in activation from the contralesional to the ipsilesional sensorimotor regions seen in several studies detailed in this review is in keeping with studies of spontaneous recovery and neurorehabilitation-related improvements in stroke [8]. The theories of interhemispheric balance after stroke posit that lesions within the motor areas of the affected ipsilesional hemisphere cause disinhibition of the contralesional motor regions [8]. The inappropriate activation of the contralesional hemisphere during movement of the affected arm is usually a maladaptive response with inhibitory influence on function; downregulation of contralesional activation is associated with improved upper limb function in stroke recovery [8].

Functional neuroimaging of NIBS in stroke recovery offers the opportunity to study the effects of a treatment modality that may provide enhanced or accelerated stroke recovery. As such, it can help delineate whether mechanisms of NIBS consist of similar factors that drive spontaneous recovery or motor learning, or whether it is via an alternative pathway. The current study suggests that the improved arm function derived from rTMS and NIBS is largely associated with a shift in interhemispheric balance.

Whilst fMRI and fNIRS both utilise the BOLD response to determine task-related cerebral activity, they each have different strengths and weaknesses. fMRI has excellent spatial resolution but is prone to movement artefact, whilst fNIRS has much lower spatial resolution and difficulties in reliability in those with denser hair and darker skin tones, but better temporal resolution and greater resistance to movement artefact [12]. Whilst modern systems of high-density diffuse optical tomography (HD-DOT) can overcome some of these challenges of traditional fNIRS, these have not yet been utilised in studies of NIBS in stroke.

In assessing the nature of fMRI/fNIRS activation as a potential biomarker of stroke recovery, it is important to consider determining causality as opposed to correlation. This approach has been discussed in studies of resting-state fMRI through consideration of the Bradford–Hill criteria [34]. The current review demonstrates the consistency of finding changes in interhemispheric balance with task fMRI after NIBS, and these findings are coherent and biologically plausible considering the current scientific understanding of stroke recovery. The consistency of these findings between two modalities (fMRI and fNIRS) strengthens the case that this is a true effect. Similarly, resting-state fMRI studies have consistently shown increased interhemispheric connectivity following rTMS [35,36] and tDCS [37,38], which is in keeping with this. Whilst one study demonstrates a potential temporal relationship between task fMRI activity post-intervention and motor outcomes at three months [25], most studies do not explore fMRI and clinical assessments at multiple time points to determine whether fMRI changes precede (and therefore potentially drive) motor improvements or whether they change in parallel. Furthermore, the studies cited do not explore a dose–response relationship with low-dose vs. high-dose designs or short-term vs. long-term use. Finally, as several studies did not have a control group of sham stimulation, these experimental paradigms do not reliably differentiate the effects due to NIBS, spontaneous recovery, rehabilitation, and placebo effect.

There is great variation in individual task-related fMRI/fNIRS activation in subacute and chronic stroke. Whilst increases in ipsilesional activation in some individuals may represent a shift from contralesional activation or restitution of function through activation in peri-infarct regions, previous studies also emphasise that a narrowing of activation from a more diffuse pattern may be a marker of good recovery [39]. As such, in some individuals, reductions in ipsilesional activation may be a marker of greater recovery. In contrast to this, in some individuals with very severe stroke, the contralesional activation may not be maladaptive and may be contributory to function [40]. This variability can make a group-level comparison more challenging. The N-of-1 approach, with individuals undergoing serial assessments and correlating clinical changes due to an intervention alongside functional neuroimaging, is the ideal approach, although it has limited scalability. This strategy also enables precision medicine approaches such as closed-loop adaptable NIBS systems that respond to individual-level feedback from fNIRS. Stratifying individuals by pattern of activation at baseline and adopting different outcome measures informed by the existing knowledge of stroke recovery is an alternative approach. Yamada et al. [28] employed this approach by assessing laterality index in those with bilateral activation patterns with motor tasks and assessing the ipsilesional activity in those with no activation/ipsilesional activation patterns.

In the included studies, neuroimaging paradigms varied widely. Task parameters, including movement frequency, pacing, complexity, and amplitude of finger tapping, can all substantially influence the nature of fMRI activation. In stroke populations, this is further compounded when physical impairment restricts movement. However, an important mitigating factor is that the included studies employed within-subject, repeated-measures designs and used identical task protocols at baseline and post-intervention.

Invasive and non-invasive VNS are emerging treatment options for post-stroke motor recovery [41,42]. Whilst no studies of VNS met the inclusion criteria, two studies have assessed the effects of tVNS on fNIRS during upper limb movements in stroke populations [43,44]. In a study of subacute stroke, tVNS delivered acutely was found to increase activation in several brain regions following a paretic hand movement task (ipsilesional postcentral gyrus, precentral gyrus, supplementary motor area, middle frontal gyrus orbital part; contralesional orbital middle frontal gyrus and dorsolateral superior frontal gyrus) compared to sham tVNS [44]. In a separate study, the acute effects of left tVNS led to differential effects in paretic arm movements from left- and right-sided stroke lesions, suggesting a potentially lateralised effect of tVNS [43]. Further studies of functional neuroimaging pre- and post-intervention with tVNS and rehabilitation are ongoing but not yet reported, including the multi-centre TRICEPS trial (ISRCTN20221867).

Across the included studies, NIBS interventions were generally well tolerated, with no treatment-related serious adverse events reported. The most commonly reported side effects were mild and transient, including tingling sensations at electrode sites for tDCS and occasional headaches with rTMS, with appropriate exclusion criteria employed to minimise risks [18,20,21,25,32].

While this review focuses on task-based functional neuroimaging, novel complementary imaging biomarkers such as quantitative susceptibility mapping (QSM) and oxygen extraction fraction (OEF) mapping may provide tissue-level structural and metabolic information, particularly within the peri-infarct regions. These could be integrated with the network-level functional data from task-based fMRI/fNIRS to comprehensively characterise NIBS-induced recovery mechanisms [45].

These findings are consistent with recent systematic reviews of neuroimaging biomarkers in stroke recovery and with resting-state NIBS studies demonstrating modulation of interhemispheric and sensorimotor network connectivity [46].

There is considerable variation in task-related fMRI/fNIRS activation patterns in individuals with subacute and chronic stroke, and the interpretation of activation changes must be contextualised by baseline patterns and stroke severity. While increases in ipsilesional activation may represent beneficial reorganisation in some patients, such as recruitment of perilesional tissue through axonal sprouting or restoration of activity in previously suppressed regions, reductions in ipsilesional activation can also indicate recovery in others. Specifically, narrowing of activation from an initially diffuse, compensatory pattern involving secondary motor areas towards more focused, efficient activation can be a marker of good recovery [47].

A limitation of the current evidence base is that several of the studies lacked sham control groups, making it difficult to distinguish between NIBS-specific neural changes and those driven by concurrent motor training, spontaneous recovery, or placebo effects. Of the seventeen included studies (rTMS only), five did not include a control intervention. Whilst this can make it difficult to infer whether the observed changes are due to spontaneous recovery over time, rehabilitation, or the rTMS intervention, the neuroimaging patterns observed in these uncontrolled studies were consistent with those from the randomised controlled trials, showing more focal activation patterns and shifts towards ipsilesional activation. Future studies employing factorial designs that systematically vary both NIBS and rehabilitation intensity would help disentangle these effects and clarify the specific mechanisms by which NIBS enhances motor recovery.

## 5. Strengths and Limitations

The current study is a comprehensive review that analyses task-related functional neuroimaging following NIBS in arm recovery after stroke and is novel in integrating rTMS and tDCS findings with task-evoked fMRI and fNIRS to provide a synthesis of their effects on motor network activation. There are several limitations to the current study. First, many included articles had no control intervention. Second, most studies were conducted in East Asia; therefore, the applicability of their findings to other populations is not clear. Third, several studies were underpowered to draw conclusions about a group-level effect. Fourth, there was heterogeneity in stroke subtypes, time post-stroke, NIBS parameters, and duration of intervention. Fifth, several rTMS studies lacked sham control groups, making it difficult to establish a causal relationship.

## 6. Conclusions

Non-invasive brain stimulation methods are promising tools for promoting arm recovery after stroke. Changes in task-related fMRI and fNIRS activation are commonly observed following NIBS and may reflect underlying neuroplastic processes. However, while functional neuroimaging shows potential as a biomarker of recovery, it is not yet sufficiently validated for routine clinical application. The current evidence is limited by small sample sizes, heterogeneity in stroke populations and NIBS parameters, and variable study quality. Future work should aim to (1) evaluate the consistency of these findings in larger, adequately powered cohorts; (2) conduct longitudinal studies with serial neuroimaging assessments at multiple time points to investigate the temporal relationship between clinical and neuroimaging outcomes and establish whether imaging changes predict subsequent recovery; (3) stratify participants by stroke severity (mild–moderate versus severe) and baseline activation patterns; and (4) employ rigorous sham-controlled designs to isolate NIBS-specific effects from spontaneous recovery and rehabilitation.

## Figures and Tables

**Table 1:** Study characteristics.

Study	Country	Type of Study	*N*	Mean Age (Years)	% Male	Time Post-stroke (Mean)	%Right Sided Stroke	Stroke Subtype	%Ischaemic Stroke	Type of Stimulation	Control Group	Concurrent Therapy	Duration of Treatment
rTMS													
Chang et al. (2012) [17]	Korea	RCT	17	58.8	59	10 months	24	Any	82	HF-rTMS	Sham rTMS	Sequential finger motor training tasks after rTMS	2 weeks
Yamada et al. (2013) [28]	Japan	Single arm	47	59.1	77	66.8 months	37.5	Any	38	LF-rTMS	None	120 min of intensive OT after rTMS.	15 days
Koganemaru et al. (2015) [30]	Japan	Single arm	11	60.8	73	31 months	45	Subcortical	64	HF-rTMS	None	Extensor training of wrist and fingers aided by peripheral neuromuscular electrical stimulation	6 weeks
Tosun et al. (2017) [20]	Turkey	RCT	25	58.5	56	51.4 days	48	Ischaemic	100	Group 1: LF-rTMS Group 2: LF-rTMS + PNES	Group 3: OT	OT for 20 sessions	4 weeks
Johnson et al. (2018) [24]	USA	RCT	3	N.S.	N.S.	N.S.	N.S.	N.S.	N.S.	LF-rTMS	Sham rTMS	Brain–computer interface training using TMS-compatible EEG.	6 weeks (3 weeks rTMS)
Du et al. (2019) [25]	China	RCT	44	55.3	80	5 days	55	Ischaemic	100	HF-rTMS (Group 1) LF-rTMS (Group 2)	Sham rTMS (Group 3)	Physiotherapy for 1 h a day	5 days
Chiu et al. (2020) [21]	USA	RCT	30	65.8	47	38 months (median)	40	Ischaemic	100	Transcranial rotating permanent magnetic stimulation (TRPMS)	Sham TRPMS	None	4 weeks
Ueda et al. (2020) [23]	Japan	Single arm	30	59.7	63	71.9 months	N.S.	Any	33	LF-rTMS	None	120 min of daily OT starting within 10 min of rTMS.	15 days
Arachchige et al. (2023) [19]	Japan	Single arm	70	63	65.7	43.5 months	50	Any	40	LF-rTMS	None	120 min of daily OT after rTMS session.	12 days
Katai et al. (2023) [29]	Japan	Single arm	30	63.6	50	55 months	16.7	Subcortical	60	LF-rTMS	None	120 min OT, twice daily, 5 days per week for 2 weeks.	14 days
Ni et al. (2023) [16]	China	RCT	33	58.6	73	N.S.	50	Any	57.5	Group TMS1: LF-rTMS + HF-rTMS Group TMS2: HF-rTMS	Sham rTMS	Bobath technique therapy, OT and AO therapy for 40–60 min a day, five days a week, for four weeks.	4 weeks
Dai et al. (2024) [26]	China	RCT	32	58.3	68.8	1.93 months	50	Any	81.3	iTBS	Robot-assisted therapy alone	Robotic-assisted therapy, 20 min sessions, five days a week for four weeks. Rehabilitation therapy for >3 h a day, five days a week for four weeks.	4 weeks
tDCS													
Lindenberg et al. (2010) [32]	USA	RCT	20	58.8	75	35.4 months	35	MCA territory ischaemic	100	Bihemispheric tDCS	Sham tDCS	Physiotherapy and OT for 60 min commenced alongside active/sham tDCS.	5 days
Nair et al. (2011) [18]	USA	RCT	14	55.8	64.2	30.5 months	42.9	Ischaemic	100	Cathodal tDCS	Sham tDCS	60 min of OT concurrent to active/sham tDCS.	5 days
Allman et al. (2016) [27]	UK	RCT	24	63.5	70.8	54.1 months	70.8	Any	N.S.	Anodal tDCS	Sham tDCS	Graded Repetitive Arm Supplementary Program (GRASP), concurrent to active/sham tDCS, one hour daily for 9 days.	9 days
Kim et al. (2023) [31]	Korea	Crossover	16	56.8	43.8	75.7 months	37.5	Any	62.5	Anodal HD-tDCS	Sham HD-tDCS (crossover)	None	10 days
Li et al. (2024) [22]	China	RCT	52	57.6	80.8	93.9 days	55.8	Any	69.2	Bihemispheric tDCS	Sham tDCS	Sensorimotor training for 40 min a day following tDCS, 5 days a week for 4 weeks.	4 weeks

Key: HF-rTMS = high-frequency repetitive transcranial magnetic stimulation; iTBS = intermittent theta-burst stimulation; LF-rTMS = low-frequency repetitive transcranial magnetic stimulation; OT = occupational therapy; RCT = randomised controlled trial; tDCS = transcranial direct current stimulation; N.S. = not stated.

**Table 2:** Summary of neuroimaging conditions used across the included studies.

Study	Intervention	Clinical Outcomes	Neuroimaging Outcomes
rTMS STUDIES			
Chang et al. (2012) [17]	HF-rTMS (10 Hz) ipsilesional M1 vs. sham, 10 sessions	Significant improvement in movement accuracy after rTMS but not sham. No difference in total JHFT scores but improved performance time in simulated feeding subtask in real rTMS group.	Sham vs. baseline: ↑ ipsilesional SMA, SMC, bilateral cerebellar hemispheres, supramarginal gyrus, putamen, insula. Real vs. baseline: ↑ ipsilesional SMA/superior parietal, bilateral caudate/thalamus, contralesional insula. Intervention × time interaction: ipsilesional SMC/thalamus, contralesional caudate.
Yamada et al. (2013) [28]	LF-rTMS (1 Hz) contralesional M1, 10 sessions	Group 1 (bilateral baseline activation): FMA-UE 49.1 ± 8.6 → 54.1 ± 7.7 (*p* < 0.001). Group 2 (unilateral/no baseline activation): FMA-UE 50.2 ± 7.9 → 54.9 ± 6.9 (*p* < 0.001).	Group 1: ↑ laterality index (0.35 → 0.49, *p* < 0.05). Group 2: ↑ activated voxels (246 → 407, *p* < 0.05).
Koganemaru et al. (2015) [30]	HF-rTMS (5 Hz) ipsilesional M1, 12 sessions	Significant increase in ROM in extension, FMA-UE, MAL and MAS post-intervention. No significant difference in active ROM in flexion.	↓ ipsilesional SMC and contralesional cingulate motor cortex activation during extensor movements. No significant difference for flexor movements.
Tosun et al. (2017) [20]	LF-rTMS (1 Hz) contralesional M1, 10 sessions (Group 1), with NMES (Group 2), therapy alone (Group 3)	Compared to baseline: Significant increase in BRS-UE, FMA-UE, UE-MI and Barthel Index in all groups. Increase in FMA-UE was >MCID of 9–10 in Groups 1 and 2 but not 3.	↑ ipsilesional M1 activation (≥25% increase) in 66.7% of participants in Group 1, 57.1% in Group 2, 42.9% in Group 3.
Johnson et al. (2018) [24]	LF-rTMS (1 Hz) contralesional M1 vs. sham (*N* = 3 total)	Compared to baseline: 2 active individuals had average 73% improvement in BBT time vs. 22% in sham participant. Compared to sham: Overall improvement in finger tracking test accuracy from baseline to follow-up.	Both active individuals: ↑ ipsilesional recruitment over time. Sham: negative laterality index at first follow-up (↑ contralesional activation).
Du et al. (2019) [25]	HF-rTMS (10 Hz) ipsilesional vs. LF-rTMS (1 Hz) contralesional vs. sham, 5 sessions each	Compared to baseline: All three groups had improved FMA-UE scores. Significant group × time interaction indicated FMA-UE scores were higher in both rTMS groups compared to sham.	All groups: ↑ ipsilesional M1 activation. Group × time interaction: HF-rTMS had ↑ ipsilesional M1/SMA vs. LF-rTMS and sham. LF-rTMS had ↓ contralesional M1 vs. HF-rTMS and sham. Positive correlation between post-intervention ipsilesional M1 activation and motor function at 3 months. No correlation between change in activation and motor improvement.
Chiu et al. (2020) [21]	HF-rTMS ipsilesional + LF-rTMS contralesional, 20 sessions	Numerical but non-significant improvements in FMA-UE, ARAT, grip strength, NIHSS, TUG velocity in active group. 6/14 (43%) participants with > 4.25 point increase in FMA-UE in active group vs. 5/15 (33%) in sham.	Compared to baseline: Greater increase in active fMRI voxels in active group (median +48.5) vs. sham (−30), *p* = 0.038. Maintained at 2nd post-treatment fMRI one month later.
Ueda et al. (2020) [23]	LF-rTMS (1 Hz) contralesional M1, 12 sessions	Compared to baseline: FMA-UE 43.2 ± 10.5 → 48.1 ± 11.0 (*p* < 0.001); natural log of mean WMFT performance time 3.2 ± 0.9 → 2.8 ± 1.1 (*p* < 0.001).	↑ ipsilesional motor cortex including SMA. ↓ frontal and parietal activation. Positive correlation between baseline laterality index and BRS for hand/fingers (r = 0.42, *p* < 0.05). No significant correlation between FMA-UE or WMFT change and laterality index change.
Arachchige et al. (2023) [19]	LF-rTMS (1 Hz) contralesional M1, 12 sessions	Compared to baseline: Significant increase in FMA-UE (40.86 ± 13.04 → 46.00 ± 12.65, *p* < 0.001). Significant increase in WMFT-FAS (41.77 ± 14.37 → 46.6 ± 14.98, *p* < 0.001).	↑ ipsilesional PMC, M1, optic radiation, hippocampus, anterior thalamic radiation and contralesional hippocampus, putamen, caudate, forceps minor. No significant deactivations.
Katai et al. (2023) [29]	LF-rTMS (1 Hz) contralesional M1, 18 sessions	Compared to baseline: Improvements in MAS at wrist (−0.22 ± 0.41, *p* = 0.009), FMA-UE (+2.53 ± 2.32, *p* < 0.001), WMFT performance time (+1.93 ± 2.39, *p* < 0.001), MAL amount of use (+0.56 ± 0.48, *p* < 0.001) and quality of movement (+0.47 ± 0.57, *p* < 0.001).	↓ contralesional M1, S1, PMC and SMA. ↑ laterality index (shift toward ipsilesional activation) in M1, S1 and PMC.
Ni et al. (2023) [16]	Combined HF + LF-rTMS (TMS1) vs. HF-rTMS (TMS2) vs. sham, 20 sessions	Compared to baseline: All groups had ↑ FMA-UE at 2 and 4 weeks. Compared to sham: Combined rTMS group had greater increases in FMA-UE scores at 2 and 4 weeks. TMS1 (HF + LF) had greater increase in FMA-UE at 4 weeks than TMS2 (HF-only).	Compared to baseline: After 4 weeks, oxygenated Hb in contralesional SMC ↓ and ipsilesional SMC ↑. Changes had higher statistical significance in combined TMS group.
Dai et al. (2024) [26]	HF-iTBS (50 Hz) ipsilesional M1 vs. control, 20 sessions	Compared to control group: Greater increase in 4-week FMA-UE score in iTBS group (39.12 vs. 35.36).	Compared to baseline: ↑ laterality index (shift toward ipsilesional) in active but not control. Compared to control: iTBS associated with greater number of activated brain regions (channels within M1 and pSMA).
tDCS STUDIES			
Lindenberg et al. (2010) [32]	Bihemispheric tDCS (1.5 mA, 30 min) vs. sham, 5 sessions	Compared to baseline: Real tDCS group FMA-UE 38.2 ± 13.3 → 43.8 ± 12.3; WMFT 0.87 ± 0.55 → 0.74 ± 0.48 at 3 days post-intervention. Compared to sham: Interaction between group and time revealed effect of time was different for FMA-UE and WMFT in active group.	For affected elbow: ↑ activation in ipsilesional M1 and PMC. For affected wrist: ↑ activation in ipsilesional M1/PMC and contralesional IFG. No significant changes in sham. No significant deactivations. Correlation between laterality index in precentral gyrus (elbow task) and WMFT improvement in real tDCS but not sham.
Nair et al. (2011) [18]	Cathodal tDCS contralesional M1 (1 mA, 30 min) vs. sham, 5 sessions	Compared to sham: 19.2% improvement in 3-joint ROM (active/passive) in active tDCS vs. 2.26% in sham at end of intervention. Group × time interaction suggesting greater increase in ROM in active tDCS. FMA-UE: +4.14 points (active) at day 7 post-intervention vs. +1.6 points (sham). Significant time × group interaction.	↓ contralesional motor region activation in 5/7 (71%) cathodal tDCS patients vs. 3/6 (50%) sham patients. Inverse correlation between ↓ contralesional activation and ↑ FMA-UE.
Allman et al. (2016) [27]	Anodal tDCS ipsilesional M1 (1 mA, 20 min) vs. sham	Compared to baseline at 3 months: Mean increase in FMA-UE not significantly different between active and sham. Mean difference in ARAT higher in active group (5.76, 95% CI 1.56–9.97, *p* = 0.045). Mean increase in WMFT scores higher in active group (6.87, 95% CI 3.41–10.33).	Compared to sham: Active tDCS group had greater increases in fMRI activation in several brain regions including ipsilesional motor areas.
Kim et al. (2023) [31]	Anodal HD-tDCS ipsilesional M1 (1 mA, 20 min) vs. sham (crossover), 5 sessions	Compared to sham HD-tDCS: No significant differences in accuracy by block × condition interaction. For response time, significantly lower by block on days 3, 4 and 5; no significant change in response time between blocks on any day.	Compared to baseline: ↑ oxygenated Hb concentration in ipsilesional motor cortex during affected hand motor task during active tDCS and from baseline to day 5 in active (but not sham).
Li et al. (2024) [22]	Bihemispheric tDCS (2 mA, 20 min) vs. sham, 20 sessions	Compared to baseline: Both groups showed significant improvements in FMA-UE, ARAT, BBT scores. Compared to sham: Active tDCS led to higher increases in mean FMA-UE, ARAT and BBT scores than sham.	Compared to sham: Significantly ↑ activation in 4 channels (10, 12, 15, 18) following treatment. These corresponded to right DLPFC, right somatosensory association cortex and right M1.

Abbreviations: ARAT, Action Research Arm Test; BBT, Box and Block Test; BRS-UE, Brunnstrom Stages of Stroke Recovery Upper Extremity; DLPFC, dorsolateral prefrontal cortex; FMA-UE, Fugl–Meyer Assessment Upper Extremity; HF, high-frequency; IFG, inferior frontal gyrus; iTBS, intermittent theta burst stimulation; JHFT, Jebsen–Taylor Hand Function Test; LF, low-frequency; M1, primary motor cortex; MAL, Motor Activity Log; MAS, Modified Ashworth Scale; MCID, minimal clinically important difference; NMES, neuromuscular electrical stimulation; NIHSS, National Institutes of Health Stroke Scale; PMC, premotor cortex; pSMA, pre-supplementary motor area; ROM, range of motion; rTMS, repetitive transcranial magnetic stimulation; S1, primary somatosensory cortex; SMA, supplementary motor area; SMC, sensorimotor cortex; tDCS, transcranial direct current stimulation; TUG, Timed Up and Go; UE-MI, Upper Extremity Motor Index; WMFT, Wolf Motor Function Test; WMFT-FAS, WMFT Functional Ability Scale; ↑, increased/improvement; ↓, decreased/reduction. Side arrows denote post-intervention changes. Alternating background colours have been applied to the table to enhance readability and do not convey additional information.

**Table 3:** Risk of Bias Assessment for RCTs of rTMS and tDCS.

Study	Randomisation Process	Deviations from Interventions	Missing Outcome Data	Outcome Measurement	Selective Reporting	Overall
rTMS						
Chang et al., 2012 [17]	Low	Some	Some	Low	Low	Some
Tosun et al., 2017 [20]	Low	Low	Low	Low	Low	Low
Johnson et al., 2018 [24]	Some	Low	Low	Low	Low	Low
Du et al., 2019 [25]	Low	Low	Low	Low	Low	Low
Chiu et al., 2020 [21]	Low	Low	Low	Low	Low	Low
Ni et al., 2023 [16]	Some	Some	Low	Some	Low	Some
Dai et al., 2024 [26]	Some	Some	Low	Low	Low	Some
tDCS						
Lindenberg et al., 2010 [32]	Low	Low	Low	Low	Low	Low
Nair et al., 2011 [18]	Low	Low	Low	Low	Low	Low
Allman et al., 2016 [27]	Low	Low	Low	Low	Low	Low
Li et al., 2024 [22]	Low	Low	Low	Low	Low	Low

**Table 4:** Risk of bias assessment for non-RCTs of rTMS and tDCS.

Study	Bias Due to Confounding	Bias in Selection of Participants	Bias in Classification of Interventions	Bias Due to Deviations from Intended Interventions	Bias Due to Missing Data	Bias in Measurement of Outcomes	Bias in Selection of Reported Results	Overall Risk of Bias
rTMS								
Yamada et al., 2013 [28]	Low	Low	Low	Low	Low	Moderate	Moderate	Moderate
Koganemaru et al., 2015 [30]	Low	Low	Low	Low	Low	Moderate	Moderate	Moderate
Ueda et al., 2020 [23]	Low	Low	Low	Low	Low	Moderate	Moderate	Moderate
Arachchige et al. 2023 [19]	Low	Low	Low	Low	Low	Moderate	Moderate	Moderate
Katai et al., 2023 [29]	Low	Low	Low	Low	Low	Moderate	Moderate	Moderate
tDCS								
Kim et al., 2023 [31]	Low	Low	Low	Low	Low	Low	Low	Low

## Data Availability

The raw data supporting the conclusions of this article will be made available by the authors on request.

## Supplementary Materials

The following supporting information can be downloaded at: https://www.mdpi.com/article/10.3390/biomedicines14010117/s1. Table S1: PRISMA 2020 Reporting Checklist Table S2: Full Electronic Search Strategy; Table S3: Expanded Version of Table 2: Summary of Neuroimaging Conditions Across Included Studies.
