# Supplementary material for: Functional Neuroimaging as a Biomarker of Non-Invasive Brain Stimulation in Upper Limb Recovery After Stroke: A Systematic Review and Narrative Discussion
Source: Biomedicines. 2026 Jan 6;14(1):117. doi: 10.3390/biomedicines14010117 (PMC12838316; doi:10.3390/biomedicines14010117)
Supplement: Supplementary file 1 [file biomedicines-14-00117-s001.zip › biomedicines-3983902-Supplimentary Table S3.pdf]

Table S3. Details of Clinical and Neuroimaging Outcomes from Included Trials.

Study	Experimental Group: Details of Stimulation	Control Group: Details of Stimulation	Clinical Assessments of Arm Function	Imaging Modality	Neuroimaging Task Details	Clinical Outcomes	Neuroimaging Outcomes
**rTMS**							
Chang et al. (2012)[17]	Ipsilesional M1, HF-rTMS, 10 Hz, 1000 pulses, 80% RMT. 10 sessions	One wing sham method	Movement accuracy and movement time during fMRI, JHFT	fMRI	Block designed sequential finger motor task. 10 x 50 second blocks.	Significant improvement in movement accuracy after rTMS but not sham. No difference in total JHFT scores but improved performance time in simulated feeding subtask in real rTMS group.	Sham rTMS vs baseline:<br>Increased activation in ipsilesional SMA, sensorimotor cortex, bilateral cerebellar hemispheres and bilateral supramarginal gyrus, putamen and insula<br><br>Real rTMS vs baseline:<br>Increased activation in ipsilesional SMA and superior parietal. lobe, bilateral caudate nucleus and thalamus, contralesional insular cortex.<br><br>Brain areas associated with a significant interaction between intervention and time:<br>Ipsilesional Sensorimotor cortex, thalamus and contralesional caudate. No significant decreases in activation.
Yamada et al. (2013)[28]	Contralesional M1 LF-rTMS at 1 Hz, 2400 pulses, 90% RMT. 10 sessions.	N/A	FMA-UE, WMFT	fMRI	Repetitive flexion-extension of the fingers at approximately 0.33 Hz, 30 second blocks, alternating sides	Increase in FMA-UE total motor score in both groups:<br>49.1 +/- 8.6 to 54.1 +/- 7.7 ($P < 0.001$) in those with bilateral fMRI activation patterns (Group 1)<br>50.2 +/- 7.9 to 54.9 +/- 6.9 points ($P < 0.001$) in those with unilateral/no fMRI activation pattern (Group	For group with bilateral fMRI activation during motor tasks (Group 1):<br>Increase in laterality index (0.35 to 0.49, $P < 0.05$).<br><br>For group with ipsilesional fMRI activation / no activation during motor tasks (Group 2):<br>Increase in activated voxels during task (246 to 407, $P < 0.05$).

https://doi.org/10.3390/biomedicines14010117

Study	Experimental Group: Details of Stimulation	Control Group: Details of Stimulation	Clinical Assessments of Arm Function	Imaging Modality	Neuroimaging Task Details	Clinical Outcomes	Neuroimaging Outcomes
						2).<br><br>Significant decreases in WMFT performance times in both groups.	
Koganemaru et al. (2015)[30]	Ipsilesional M1 HF-rTMS at 5 Hz at 100% active motor threshold for 8 seconds. 15 cycles of rTMS delivered immediately following 50 seconds of wrist and finger extension aided by peripheral electrical stimulation of extensor digitorum communis. 12 sessions.	N/A	Active and passive ROM in the wirst and MCP joints, MAS, pinch and grip power, FMA-UE.	fMRI	Alternating movement and rest blocks lasting 21 seconds. Movement blocks consist of either active extensor contraction or active flexor contraction of the wrist and digits at 0.25 Hz.	Significant increase in ROM in extension, FMA-UE, MAL and MAS post-intervention. No significant difference in active rOM in flexion.	Compared to baseline:<br>For extensor movements, there was a reduction in activation in ipsilesional sensorimotor cortex and contralesional cingulate motor cortex. No significant difference for flexor movements.
Tosun et al. (2017)[20]	Group 1: Contralesional M1 LF-rTMS, 1 Hz, 90% RMT, 1200 pulses. 10 sessions.<br><br>Group 2: As above plus NMES to wrist extensors for 20 sessions.	Group 3:<br>No rTMS (therapy alone).	BRS, FMAE-UE, UE-MI, MAS	fMRI	Alternating movement and rest blocks of 30 seconds repeated 5 times. Motor task consisted of unilateral flexion and extension of the fingers.	Compared to baseline:<br>Significant increase in BRS-UE, FMA-UE, UE-MI and Barthel Index in all groups. Increase in FMA-UE was > MCID of 9-10 in Groups 1 and 2 but not 3.	Compared to baseline:<br>Increased activation in the ipsilesional M1 (defined as a 25% increase in activation) in 66.7% of participants in Group 1, 57.1% in Group 2 and 42.9% in Group 3.

https://doi.org/10.3390/biomedicines14010117

Study	Experimental Group: Details of Stimulation	Control Group: Details of Stimulation	Clinical Assessments of Arm Function	Imaging Modality	Neuroimaging Task Details	Clinical Outcomes	Neuroimaging Outcomes
Johnson et al. (2018)[24]	Contralesional M1, LF-rTMS , 1Hz, 90% RMT	Sham coil (mimicked sound of coil)	BBT, Finger Tracking Test	fMRI	30 second task and rest blocks. Task was the Finger Tracking Test.	Compared to baseline:<br>2 individuals in active group had an average of 73% improvements in Box and Block test time from baseline to first follow-up whilst the sham rTMS participant had a 22% improvement.<br><br>Compared to sham rTMS:<br>An overall improvement in finger tracking test accuracy from baseline to follow-up.	Compared to baseline:<br>Boht individuals receiving active rTMS showed increased recrutiment of ipsilesional areas over time. The sham rTMS subject had a negative laterality index at first follow-up (indicating increased contralesional activation).
Du et al. (2019)[25]	Group 1:<br>Ipsilesional M1 HF-rTMS, 10 Hz, 1200 pulses at 100% RMT. 5 sessions.<br><br>Group 2:<br>Contralesional M1 LF-rTMS, 1200 pulses at 100% RMT. 5 sessions.	Sham rTMS delivered with coil rotated 90 degrees away from scalp.	FMA-UE, MRC.	fMRI	20 second blocks of affected and then unaffected hand finger tapping (finger to thumb) at 1 Hz. 5 blocks total.	Compared to baseline: All three groups had improved FMA-UE scores. A significant group-time interaction indicated FMA-UE scores were higher in both rTMS groups compared to sham.	Compared to baseline:<br>All three groups showed increase in ipsilesional M1 activation.<br><br>Group-time interaction:<br>HF-rTMS had increased activation in ipsilesional M1 and SMA compared to LF-rTMS and sham. LF-rTMS had reduced activation in contralesional M1 compared to HF-rTMS and sham.<br><br>Positive correlation between post-intervention fMRI activation in ipsilesional M1 and motor function at 3 months. No correlation between rTMS induced change in neural

Study	Experimental Group: Details of Stimulation	Control Group: Details of Stimulation	Clinical Assessments of Arm Function	Imaging Modality	Neuroimaging Task Details	Clinical Outcomes	Neuroimaging Outcomes
							activity and motor improvement from baseline to post-intervention.
Chiu et al. (2020)[21]	HF (5 Hz) Ipsilesional microstimulators in the lateral premotor cortical site, supplementary motor cortical site, ipsilesional sites surrounding infarct lesion on the premotor cortex and postcentral gyrus (in subcortical infarcts these were placed over MRI-localized precentral gyrus sites 1 and 4cm lateral to midline). LF (0.2 Hz) Contralesional microstimulators at primary motor cortical sites 1 and 4cm lateral to midline.<br><br>20 sessions.	Details not specified.	FMA-UE, ARAT, Grip strength, Pinch strength	fMRI	14 seconds of hand grip (alternating left and right) with 14 second rest intervals. 5 on and off epochs.	Numerical but non significant improvements in FMA-UE, ARAT, grip strength, NIHSS, TUG velocity in active group.<br><br>6/14 (43%) participants with > 4.25 point increase in FMA-UE in active group vs 5/15 (33%) in sham.	Compared to baseline:<br>Greater increase in the number of active fMRI voxels in the active group (median + 48.5) compared to sham (-30), p = 0.038. Maintained at 2nd post-treatment fMRI one month later.
Ueda et al. (2020)[23]	Contralesional M1 LF-rTMS at 1 Hz, 2400 pulses, 90% RMT. 12 sessions.	N/A	FMA-UE, WMFT, BRS	fMRI	Repetitive unilateral flexion-extension of the fingers at approximately 0.33 Hz, 30 second blocks, repeated 3 times.	Compared to baseline:<br>FMA-UE increased from 43.2 +/- 10.5 to 48.1 +/- 11.0 (p < 0.001) and the natural log of mean WMFT performance time decreased from 3.2 +/- 0.9 to 2.8 +/- 1.1 (p < 0.001)	Increased activation in the ipsilesional motor cortex including SMA following intervention. Reductions in frontal and parietal activation after intervention.<br><br>Positive correlation between laterality index before intervention and BRS for hand/fingers (r = 0.42, p < 0.05). No

https://doi.org/10.3390/biomedicines14010117

Study	Experimental Group: Details of Stimulation	Control Group: Details of Stimulation	Clinical Assessments of Arm Function	Imaging Modality	Neuroimaging Task Details	Clinical Outcomes	Neuroimaging Outcomes
							significant correlation between FMA-UE or WMFT and change in laterality index.
Arachchige et al. (2023)[19]	Contralesional M1 LF-rTMS at 1 Hz, 1200 or 2400 pulses, 90% RMT. 12 sessions.	N/A	FMA-UE, WMFT	fMRI	Repetitive unilateral flexion-extension of the fingers at approximately 0.33 Hz, 30 second blocks, repeated 3 times.	Compared to baseline: Significant increase in FMA-UE (40.86 +/- 13.04 to 46.00 +/- 12.65, P < 0.001). Significant increase in WMFT-FAS (41.77 +/- 14.37 to 46.6 +/- 14.98, P < 0.001)	Compared to baseline: Increased activations in ipsilesional PMC, M1, optic radiation, hippocampus, anterior thalamic radiation and contralesional hippocampus, putamen, caudate, forceps minor (corpus callosum). No significant deactivations.
Katai et al. (2023)[29]	Contralesional M1 LF-rTMS, 1 Hz, 1200 pulses, 90% RMT. 18 sessions.	N/A	BRS, MAS, FMA-UE, WMFT, ARAT, Grip Strength	fMRI	Unilateral flexion-extension of fingers at 0.5 Hz. Alternating 40 second task and rest blocks for 3 cycles on each hand.	Compared to baseline: Improvements in MAS at the wrist (- 0.22 +/- 0.41, p = 0.009), FMA-UE (2.53 +/- 2.32, p < 0.001), WMFT performance time (1.93 +/- 2.39, p < 0.001) and MAL amount of use (0.56 +/- 0.48, p < 0.001) and quality of movement (0.47 +/- 0.57, p < 0.001) scales.	Compared to baseline: Relative decrease in activation of contralesional M1, S1, PMC and SMA. Increased in laterality index (indicating shift towards more ipsilesional activation) in M1, S1 and PMC.
Ni et al. (2023)[16]	Group TMS1 (if RMT <40%): Ipsilesional M1 HF-rTMS, 10 Hz, 1200 pulses at 100% RMT plus Contralesional M1 LF-rTMS at 1 Hz, 1200 pulses at 100% RMT. 20 sessions.	Sham rTMS delivered with coil perpendicular to the skull	FMA-UE	fNIRS	Finger-nose task repeated four times on each arm (motor imagery task if unable to complete the task).	Compared to baseline: All groups had an increase in FMA-UE at 2 weeks and 4 weeks.<br><br>Compared to sham rTMS group: The combined rTMS group had greater	Compared to baseline: After 4 weeks of treatment, the oxygenated haemoglobin content in SMC area of contralesional hemisphere decreased and increased in the SMC area of the ipsilesional hemisphere. The changes had a higher level of statistical significance in the combined TMS group.

https://doi.org/10.3390/biomedicines14010117

Study	Experimental Group: Details of Stimulation	Control Group: Details of Stimulation	Clinical Assessments of Arm Function	Imaging Modality	Neuroimaging Task Details	Clinical Outcomes	Neuroimaging Outcomes
	Group TMS2 (if RMT >40%): Ipsilesional M1 HF-rTMS, 10 Hz, 1200 pulses at 100% RMT. 20 sessions.					increases in FMA-UE scores at 2 and 4 weeks. The TMS1 group (HF-rTMS + LF-rTMS) had a greater increase in FMA-UE at 4 weeks than TMS2 group (HF-rTMS).	
Dai et al. (2024)[26]	Ipsilesional M1 HF-iTBS at 50 Hz, 600 pulses at 80% AMT. 20 sessions.	N/A	FMA-UE	fNIRS	20 second task blocks alternating with 20 second rest blocks, repeated 5 times. The motor task was upward elbow flexion and shoulder flexion.	Compared to control group: Greater increase in 4 week FMA-UE score in iTBS group (39.12 vs 35.36).	Compared to baseline: Increase in laterality index (shift towards ipsilesional brain activation) in active group but not control group. Compared to control group: iTBS associated with a greater number of activated brain regions after treatment (channels within the M1 and pSMA region).
**tDCS**							
Lindenberg et al. (2010)[32]	Ipsilesional M1 anodal stimulation and contralesional M1 cathodal stimulation, 1.5mA, 30 mins. 5 sessions.	Current ramped up to 1.5mA then decreased over 30 seconds.	FMA-UE, WMFT	fMRI	35 second task and rest blocks. Tasks included elbow flexion-extension or wrist flexion-extension at 1 Hz.	Compared to baseline: Real tDCS group had an increase in FMA-UE score (38.2 +/- 13.3 to 43.8 +/- 12.3) and decrease in WMFT scores from 0.87 +/- 0.55 to 0.74 +/- 0.48 at the 3 days post-intervention time point. Compared to sham tDCS: Interaction between	Compared to baseline: For affected elbow movements - significant increase of activation in a cluster in the ipsilesional primary motor cortex and premotor cortex. For affected wrist movements - a significant increase of activation in a cluster in the ipsilesional primary motor cortex, premotor cortex and contralesional inferior frontal gyrus. No significant changes in the sham group. No significant deactivations. Correlation between laterality index in

https://doi.org/10.3390/biomedicines14010117

Study	Experimental Group: Details of Stimulation	Control Group: Details of Stimulation	Clinical Assessments of Arm Function	Imaging Modality	Neuroimaging Task Details	Clinical Outcomes	Neuroimaging Outcomes
						group and time revealed that the effect of time was different for FMA-UE and WMFT in the active group.	precentral gyrus following the elbow movement task and WMFT performance improvement in the real tDCS group but not sham.
Nair et al. (2011)[18]	Contralesional M1 cathodal tDCS at 1mA for 30 mins. 5 sessions.	Sham tDCS - turning current up and then turning off.	ROM for shoulder abduction, elbow extension and wrist extension; FMA-UE.	fMRI	35 seconds task and rest blocks, repeated 5 times for each task. Motor tasks included wrist flexion-extension and elbow flexion-extension at 1 Hz.	Compared to sham tDCS: 19.2% improvement in 3 joint range of motion (active/passive ROM) in active tDCS vs 2.26% in sham tDCS at the end of the interventionA group-time interaction suggesting the effect of time was different for active tDCS compared to sham post-intervention (i.e. greater increase in ROM in the active tDCS group).<br><br>A 4.14 point increase in FMA-UE at day 7 post-intervention compared to baseline versus a 1.6 point increase for sham tDCS. A significant time-group interaction suggesting the effect of time was different between the groups (i.e. greater increase in	The magnitude of activation in the contralesional motor region decreased in 5/7 (71%) patients in cathodal tDCS group compared to 3/6 (50%) patients in the sham group. An inverse correlation was found between decreases in contralesional activation and increases in FMA-UE.

https://doi.org/10.3390/biomedicines14010117

Study	Experimental Group: Details of Stimulation	Control Group: Details of Stimulation	Clinical Assessments of Arm Function	Imaging Modality	Neuroimaging Task Details	Clinical Outcomes	Neuroimaging Outcomes
						FMA-UE in active tDCS group).	
Allman et al. (2016)[27]	Ipsilesional M1 anodal tDCS at 1mA for 20 mins	Sham tDCS - ramping current up to 1mA over 10 seconds then turning off.	FMA-UE, ARAT, WMFT.	fMRI	30 second task and rest blocks. Passive flexion-extension of the hand by a researcher at 1 Hz.	Compared to baseline: At 3 months, the mean increase in FMA-UE was not significantly different between active and sham groups. The mean difference in ARAT was higher in the active group (5.76, 95% CI 1.56 to 9.97, p = 0.045). The mean increase in WMFT scores were higher in the active group (6.87, 95% CI 3.41 to 10.33).	Compared to sham tDCS: The active tDCS group had greater increases in fMRI activation in several brain regions including ipsilesional motor areas.
Kim et al. (2023)[31]	Ipsilesional motor cortex anodal HD-tDCS, 1mA for 20 mins. 5 sessions.	Crossover: Sham tDCS with 30s of HD-tDCS followed by ramping down current. 5 sessions.	Accuracy and response time during sequential finger tapping task.	fNIRS	Sequential finger tapping task sequence consisting of 9 digits. 15 blocks of the same sequence.	Compared to sham HD-tDCS: No significant differences in accuracy by block x condition interaction. For response time, the response time was significantly lower by block on day 3, 4 and 5; no significant change in response time between blocks on any day.	Compared to baseline: Increase in oxygenated Hb concentration during affected hand motor task in the region of the ipsilesional motor cortex during active tDCS and from baseline to day 5 in the active tDCS (but not sham tDCS).
Li et al. (2024)[22]	Bihemispheric tDCS with anodal, ipsilesional primary	Sham tDCS with current turned on for	FMA-UE, ARAT, BBT, Erasmus-MC revised Nottingham	fNIRS	15 second task and 20 seconds rest blocks for	Compared to baseline: Both groups showed significant improvements in	Compared to sham tDCS: Significantly increased activation in 4 channels (10, 12, 15, 18) following treatment.

Study	Experimental Group: Details of Stimulation	Control Group: Details of Stimulation	Clinical Assessments of Arm Function	Imaging Modality	Neuroimaging Task Details	Clinical Outcomes	Neuroimaging Outcomes
	somatosensory (PSC) stimulation and cathodal contralesional PSC stimulation at 2mA for 20 mins. 20 sessions.	30 seconds then ramped down.	Sensory Assessment Scale, Neurometer sensory quantitative detector		3 cycles. Motor task was bilateral hand clenching and unclenching,	FMA-UE, ARAT, BBT scores.<br><br>Compared to sham tDCS: Active tDCS led to higher increases in mean FMA-UE, ARAT and BBT scores than sham.	These corresponded to the right dorsolateral prefrontal cortex, right somatosensory association cortex and right primary motor cortex.

Table 2 Key: ARAT – Action Research Arm Test; BBT – Box and Block Test; FMA-UE – Fugl-Meyer Assessment – Upper Extremity; fMRI – Functional Magnetic Resonance Imaging; fNIRS – Functional Near-Infrared Spectroscopy; M1 – Primary Motor Cortex; MRC – Medical Research Council; PMC – Premotor Cortex; S1 – Primary Somatosensory Cortex; SMA – Supplementary Motor Area; SMC – Supplementary Motor Cortex; tDCS – Transcranial Direct Current Stimulation; WMFT – Wolf Motor Function Test.

https://doi.org/10.3390/biomedicines14010117
